# Post-translational modification and mitochondrial function in Parkinson’s disease

**DOI:** 10.3389/fnmol.2023.1329554

**Published:** 2024-01-11

**Authors:** Shishi Luo, Danling Wang, Zhuohua Zhang

**Affiliations:** ^1^Institute for Future Sciences, Hengyang Medical School, University of South China, Hengyang, Hunan, China; ^2^Key Laboratory of Rare Pediatric Diseases, Ministry of Education, Hengyang, Hunan, China; ^3^The Affiliated Changsha Central Hospital, Hengyang Medical School, University of South China, Changsha, Hunan, China; ^4^Institute of Molecular Precision Medicine, Xiangya Hospital, Key Laboratory of Molecular Precision Medicine of Hunan Province and Center for Medical Genetics, Hunan Key Laboratory of Medical Genetics, Central South University, Changsha, Hunan, China

**Keywords:** Parkinson’s disease, post-translational modification (PTM), mitochondrial function, ubiquitination, phosphorylation, acetylation, SUMOylation, s-nitrosylation

## Abstract

Parkinson’s disease (PD) is the second most common neurodegenerative disease with currently no cure. Most PD cases are sporadic, and about 5–10% of PD cases present a monogenic inheritance pattern. Mutations in more than 20 genes are associated with genetic forms of PD. Mitochondrial dysfunction is considered a prominent player in PD pathogenesis. Post-translational modifications (PTMs) allow rapid switching of protein functions and therefore impact various cellular functions including those related to mitochondria. Among the PD-associated genes, *Parkin*, *PINK1*, and *LRRK2* encode enzymes that directly involved in catalyzing PTM modifications of target proteins, while others like α-synuclein, FBXO7, HTRA2, VPS35, CHCHD2, and DJ-1, undergo substantial PTM modification, subsequently altering mitochondrial functions. Here, we summarize recent findings on major PTMs associated with PD-related proteins, as enzymes or substrates, that are shown to regulate important mitochondrial functions and discuss their involvement in PD pathogenesis. We will further highlight the significance of PTM-regulated mitochondrial functions in understanding PD etiology. Furthermore, we emphasize the potential for developing important biomarkers for PD through extensive research into PTMs.

## 1 Introduction

Parkinson’s disease (PD) is the most common neurodegenerative movement disease, affecting more than 10 million people worldwide (Kalia and Lang, 2015). Pathologically, PD is characterized by the progressive loss of dopaminergic (DA) neurons in the substantia nigra pars compacta (SNpc) and the accumulation of aggregated α-synuclein in the form of intracellular inclusion called Lewy Body (LB) (Tanner, 1992). The manifestations of PD are chronic and progressive, with the main symptoms involving movement dysfunctions such as tremor, tonicity, bradykinesia, and postural instability. Many patients also experience prodromal symptoms, including non-motor disturbances such as constipation, hyposmia, and mood disorders (Martinez-Martin et al., 2007; Lajoie et al., 2021). Currently, there is no cure for PD. The available treatments only alleviate the movement symptoms with little effects on disease progression.

Most PD cases are sporadic, or idiopathic, with age and environmental exposures (such as pesticides, herbicides, heavy metal, and head injury) being the main known risk factors (Goldman, 2014; Ascherio and Schwarzschild, 2016). About 15% PD cases have a familial history, and about 5–10% of PD patients present a monogenic inheritance pattern (Hernandez et al., 2016). Mutations in at least 20 genes are identified to be linked with familiar PD (Table 1). From which, dominantly associated genes such as *SNCA* (α-*synuclein*), *LRRK2*, and *VPS35*, as well as recessively associated genes like *PRKN* (*Parkin*), *DJ-1*, *GBA*, *PINK1*, *ATP13A2*, and *FBXO7*, are identified (Polymeropoulos et al., 1997; Kitada et al., 1998; Bonifati et al., 2003; Lwin et al., 2004; Valente et al., 2004; Gilks et al., 2005; Ramirez et al., 2006; Di Fonzo et al., 2009; Vilarino-Guell et al., 2011; Funayama et al., 2015). More than additional 90 genetic risk loci are shown to be associated with idiopathic PD (Bekris et al., 2010; Nalls et al., 2019). Individuals with genetic changes of those genes likely predispose to PD.

**TABLE 1:** Genes associated with familiar PD.

	Gene	PARK locus	Alternative names	Inheritance	Type of parkinsonism
Widely validated	*SNCA*	*PARK1*, *PARK4*	*NCAP*	AD	Early/late onset, atypical
	*PRKN*	*PARK2*	*Parkin*	AR	Early onset, typical
	*PINK1*	*PARK6*	*BRPK*	AR	Early onset, typical
	*DJ-1*	*PARK7*	*GATD2*	AR	Early onset, typical
	*LRRK2*	*PARK8*	*ROCO2*, *RIPK7*	AD	Late onset, typical
	*ATP13A2*	*PARK9*	*HSA9947*, *CLN12*	AR	Juvenile onset, atypical
	*PLA2G6*	*PARK14*	*PNPLA9*, *IPLA2*	AR	Juvenile onset, atypical
	*FBXO7*	*PARK15*	*FBX7*	AR	Early onset, atypical
	*VPS35*	*PARK17*	*MEM3*	AD	Late onset, typical
	*DNAJC6*	*PARK19*	*KIAA0473*, *DJC6*	AR	Juvenile onset, atypical
	*SYNJ1*	*PARK20*	*INPP5G*	AR	Juvenile onset, atypical
	*DNAJC13*	*PARK21*	*KIAA0678*, *RME8*	AD	Late onset, typical
	*VPS13C*	*PARK23*	*KIAA1421*, *BLTP5C*	AR	Early onset, atypical
	*POLG*	-	*POLG1*, *POLGA*	AD	Early onset, atypical
Less validated	*UCHL1*	*PARK5*	*PGP9.5*	AD	Late onset, typical
	*GIGYF2*	*PARK11*	*TNRC15*, *PERQ2*, *GYF2*	AD	Late onset, typical
	*HTRA2*	*PARK13*	*OMI*	AD	Late onset, typical
	*EIF4G1*	*PARK18*	*P220*	AD	Late onset, typical
	*CHCHD2*	*PARK22*	*C7orf17*, *MIX17B*	AD	Late onset, typical
	*PSAP*	*PARK24*	*GLBA*, *SAP1*	AD	Late onset, typical

AD, autosomal dominant; AR, autosomal recessive; juvenile-onset, clinical symptom starting before 21 years; early-onset, clinical symptom starting between 20 and 60 years; late-onset, clinical symptom starting after 60 years; typical PD, PD cases presenting classical motor symptoms, with slow progression and good response to dopaminergic therapeutics; atypical PD, PD cases featuring prominent additional neurological signs, such as dementia, spasticity, dystonia, with or without abnormal ocular movements (Cherian and Vijayaraghavan, 2023); “-” means no answer.

Human genetics have significantly contributed to our understanding of the molecular mechanisms of PD pathogenesis. Several key pathways, including those related to protein misfolding and aggregation, the ubiquitin-proteasomal system, autophagy, mitochondrial dysfunction, lysosomal abnormality, and vesicle trafficking, are revealed to PD (Henchcliffe and Beal, 2008; Meredith et al., 2009; Ebrahimi-Fakhari et al., 2012; Hunn et al., 2015; De Virgilio et al., 2016; Blumenreich et al., 2020). Of particular, mitochondrial dysfunction has long been implied in PD pathogenesis. Methyl-4-phenyl-1,2,3,4-tetrahydropyridine (MPTP), a mitochondrial complex I inhibitor, induces Parkinsonism in both human and animals (Langston et al., 1983). Multiple pesticides and herbicides are shown to induce parkinsonism via mitochondria-mediated mechanisms (Chen et al., 2017). A number of PD pathogenic monogenetic genes either encode mitochondrial proteins or regulate mitochondrial functions (Nicoletti et al., 2021). Genome-wide association studies (GWAS) also indicate that mitochondria-related processes are involved in both familiar and sporadic forms of PD (Billingsley et al., 2019).

Post-translational modifications (PTMs) refer to covalent chemical alterations of a protein after its synthesis. More than 400 types of PTMs are identified (Khoury et al., 2011). Some widely studied PTMs include phosphorylation, ubiquitination, methylation, acetylation, SUMOylation, etc. PTMs increase functional diversity of the modified protein, therefore modulate almost every aspect of cellular processes, including mitochondrial functions (Karve and Cheema, 2011; Stram and Payne, 2016). Among the PD-associated genes, *Parkin*, *PINK1*, and *LRRK2* encode enzymes that directly catalyze the PTM of target proteins while they are PTM modified themselves (Trempe et al., 2013; Eiyama and Okamoto, 2015; Taylor and Alessi, 2020). PD-related proteins like α-synuclein, FBXO7, HTRA2, VPS35, CHCHD2, and DJ-1 either participate in or are heavily modified by PTMs (Mitsumoto et al., 2001; Plun-Favreau et al., 2007; Kang et al., 2012; Burchell et al., 2013; Tang et al., 2015; Meng et al., 2017). In this review, we aim to summarize the recent findings on major PTMs associated with PD-related proteins, either as enzymes or substrates, that have been shown to play significant roles in various mitochondrial functions, hence, PD pathogenesis.

## 2 Ubiquitination: regulating mitochondrial functions in Parkinson’s disease

Ubiquitination is a PTM characterized by the covalent binding of ubiquitin (Ub) molecule to a specific target protein. This essential PTM primarily determines protein stability by marking the target protein for proteasomal degradation. However, it has also been reported to be able to enhance protein stability and regulate protein activities. Ubiquitination plays an essential role in maintaining normal mitochondrial functions. Dysregulated ubiquitination can cause mitochondrial dysfunctions. While the accumulation of misfolded and ubiquitinated α-synuclein has long been recognized as the toxic factor in PD brain pathology, evidence implicating the direct involvement of ubiquitination dysregulation in mitochondrial dysfunction and thus PD pathology emerged from the intensive study of PD associated Parkin and PINK1. As non-mitochondrial proteins, other PD-related proteins, such as VPS35, FBXO7, and LRRKs, are found to regulate mitochondrial functions by modifying ubiquitination process (Table 2).

**TABLE 2:** Ubiquitination of PD-related proteins and mitochondrial dysfunction.

PD-related protein		Enzyme	Substrate	Regulated mitochondrial function	References
Parkin	Directly involved in PTM enzymatic reaction	Parkin	Parkin	Activates mitophagy	Zhang et al., 2000
		Parkin	OMM proteins	Activates mitophagy	Gegg et al., 2010; Lazarou et al., 2012; Sun et al., 2012; Ordureau et al., 2015; Liu et al., 2018; Lopez-Domenech et al., 2018
		Parkin	Tollip	Promotes MDVs transport	Ryan et al., 2020
		Parkin	SNX9	Inhibits immune-related-MDVs formation	Matheoud et al., 2016
		Parkin	DRP1	Inhibits mitochondrial fission	Wang H. et al., 2011
		Parkin	MFN1	Inhibits mitochondrial fusion	Gegg et al., 2010
		Parkin	MFN2	Inhibits mitochondrial fusion, increases mitochondria-ER contacts	Gegg et al., 2010; Basso et al., 2018
		Parkin	PARIS	Promotes mitochondrial biogenesis	Siddiqui et al., 2016
		Parkin	MICU1	Maintains calcium homeostasis	Matteucci et al., 2018
		Parkin	MIRO	Increases mitochondria-ER contacts	Wang X. et al., 2011
α-synuclein		SIAH-1	α-synuclein	Increases cytochrome c release	Lee et al., 2008
FBXO7		FBXO7	PINK1	Increases mitophagy	Liu et al., 2020
VPS35	Indirectly involved in PTM enzymatic reaction	MULAN	MFN2	Promotes mitochondrial fusion	Tang et al., 2015
LRRK2		PERK	MARCH5, MULAN, Parkin	Inhibits mitochondria-ER contacts	Toyofuku et al., 2020

Tollip, the endosomal adaptor Toll interacting protein; SNX9, sorting nexin 9; DRP1, dynamin-related protein 1; MFN1 and 2, mitofusin 1 and 2; PARIS, Parkin-interacting substrate; MICU1, mitochondrial calcium uptake 1; MIRO, mitochondrial Rho GTPase; PINK1, PTEN induced putative kinase 1; MULAN, mitochondrial E3 ubiquitin protein ligase 1; LRRK2, leucine-rich repeat kinase 2; MARCH5, membrane associated Ring-CH-type finger 5; ER, endoplasmic reticulum; MDV, mitochondrial-derived vesicle.

### 2.1 Parkin-mediated ubiquitination and mitophagy

Mutations in *Parkin* gene are the most common genetic cause of juvenile-onset and early-onset PD (EOPD), defined by the appearance of PD symptoms in teens and before the age of 60 (Kitada et al., 1998). The *Parkin* gene encodes Parkin, an E3 ubiquitin ligase belonging to the RING-between-RING (RBR) family, which accepts Ub from the E2 enzyme and transfers it onto the target protein (Imai et al., 2000; Shimura et al., 2000; Zhang et al., 2000). By ubiquitinating various substrates, Parkin plays a central role in maintaining mitochondrial function and homeostasis (Palacino et al., 2004; Narendra et al., 2008).

**FIGURE 1:**
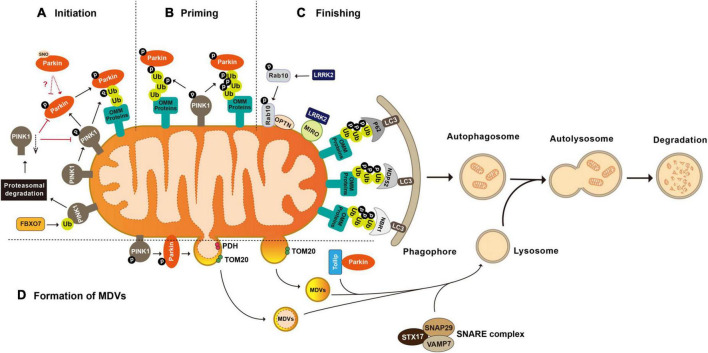
Post-translational modifications in the regulation of mitophagy and mitochondrial-derived vesicles formation. **(A)** Initiation of mitophagy. Parkin is activated and translocated onto the damaged mitochondria. PINK1 mediated phosphorylation of Parkin and mitochondria situated Ub is essential for Parkin activation. FBXO7 negatively regulates mitophagy activity by enhancing the ubiquitination of PINK1. **(B)** Priming of mitophagy. After activation, Parkin ubiquitinates a large number of mitochondrial proteins, resulting in increased Ub signal and successively increased pUb signals mediated by PINK1. **(C)** Finishing of mitophagy. Ubiquitination of OMM proteins further promotes the recruitment of Ub binding autophagy receptors such as P62/SQSTM1, OPTN, NDP52/CALCOCO2, and NBR1 to target the damaged mitochondria to LC3 positive phagophores for lysosomal degradation. LRRK2 mediated phosphorylation regulates mitophagy through its substrates MIRO and Rab10. **(D)** Formation of mitochondrial-derived vesicles (MDVs). Parkin mediated ubiquitination and PINK1 mediated phosphorylation are required by the formation of PDH+ MDVs. Parkin and Tollip facilitate the transport of TOM20+ MDVs to the endo lysosomal compartment with the help of the STX17 SNAP29 VAMP7 SNARE complex. FBXO7, F-box protein 7; PINK1, PTEN induced putative kinase 1; Ub, ubiquitin; P62/SQSTM 1, sequestosome 1; NDP52/CALCOCO2, calcium binding and coiled coil domain containing protein 2; NBR1, neighbor of Brca1; OPTN, optineurin; MIRO, mitochondrial Rho GTPase; LRRK2, leucine-rich repeat kinase 2; LC3, microtuble-associated protein light chain 3; PDH, pyruvate dehydrogenase; and TOM20, translocases of the outer membrane 20.

The groundbreaking study illustrating Parkin’s role in mitophagy, the biological pathway that selectively eliminates defective mitochondria, was conducted by the Youle laboratory (Narendra et al., 2008). Later studies collectively reveal that Parkin-mediated mitophagy consists of three major steps: (1) initiation, the activation and translocation of Parkin onto the damaged mitochondria (Narendra et al., 2008; Matsuda et al., 2010); (2) priming, the cascade ubiquitination of various targets on the outer mitochondrial membrane (OMM) by Parkin (Tanaka et al., 2010; Chan et al., 2011; Sarraf et al., 2013); and (3) finishing, the lysosomal sequestration and degradation of heavily ubiquitinated mitochondria (Karbowski and Youle, 2011). Evidence suggests that the recruitment of Parkin to the damaged mitochondria is mediated by the binding of Parkin to the mitochondria-situated phosphorylated-Ub (phospho-Ub, pUb), which is formed by the kinase activity of PINK1 (PTEN-induced putative kinase 1) (Xiong et al., 2009; Kane et al., 2014; Sauve et al., 2015). During the priming stage, Parkin ubiquitinates multiple proteins, showing a preference for OMM proteins including Parkin itself, MFN1 and 2 (mitofusin 1 and 2), MIRO1 and 2 (mitochondrial Rho GTPase 1 and 2), VDAC1-3 (voltage-dependent anion channel 1–3), CISD1 (CDGSH iron-sulfur domain 1), and TOM 20, 40, and 70 (translocases of the outer membrane 20, 40, and 70) (Gegg et al., 2010; Lazarou et al., 2012; Sun et al., 2012; Ordureau et al., 2015; Liu et al., 2018; Lopez-Domenech et al., 2018). Ubiquitination of OMM proteins further promotes the recruitment of Ub-binding autophagy receptors such as P62/SQSTM1 (sequestosome 1), OPTN (optineurin), NDP52/CALCOCO2 (calcium-binding and coiled-coil domain-containing protein 2), and NBR1 (neighbor of Brca1). In turn, these receptors elicit the targeting of the damaged mitochondria to LC3-positive phagophores for lysosomal degradation (Figure 1; Heo et al., 2015; Ordureau et al., 2018; Padman et al., 2019).

Unlike many other E3 ligases that exhibit stringent substrate specificity, Parkin appears to be loose on substrate selectivity. In addition to the well-studied Parkin substrates, recent proteomic analyses have revealed that Parkin ubiquitinates an extensive large number of proteins (Rose et al., 2016; Martinez et al., 2017). After treatment with the mitochondrial uncoupler carbonyl cyanide m-chlorophenylhydrazone (CCCP) for extended hours, most OMM proteins are ubiquitinated without necessarily having functional consequences (Chan et al., 2011; Sarraf et al., 2013). In general, Parkin is activated by PINK1-mediated phosphorylation of its N-terminal ubiquitin-like domain. With the help of PINK1, ubiquitination on the OMM by Parkin leads to more pUb and consecutively greater Parkin recruitment and activation, creating a feed-forward loop to amplify the Parkin-mediated ubiquitination to the maximum (Ordureau et al., 2014). Using artificial mitochondria-targeted proteins, Koyano et al. (2019) have found that the substrate specificity of Parkin is not determined by the amino-acid sequence within the substrate, but rather by the presence of pUb on the target protein (Durcan et al., 2012).

Proteomic analyses of purified mitochondria have revealed that Parkin produces a mixture of canonical and non-canonical Ub chains on damaged mitochondria. In general, Ub chains can form through any of the seven lysine (K6, K11, K27, K29, K33, K48, and K63) and the N-terminal methionine (Met1) (Kulathu and Komander, 2012; Akutsu et al., 2016). *In vitro*, Parkin ubiquitinates mitochondrial proteins with K6-, K11-, K48-, and K63-linked Ub chains to signal damaged mitochondria for mitophagy (Ordureau et al., 2014). Canonical K48-linked Ub chains are crucial for the proteasomal targeting and degradation of modified proteins (Manohar et al., 2019), while K63-linked Ub chains activate the autophagic machinery through recruiting autophagy adaptors like HDAC6 (histone deacetylase 6) and P62 (Seibenhener et al., 2004; Olzmann et al., 2007). Intriguingly, Parkin catalyzes certain degrees of K6- and K11-linked Ub chains on OMM proteins. Activity of Parkin-mediated mitophagy is impaired when either K6 or K11 Ub-linkage was inhibited by the expression of mutant Ub K6R or K11R, suggesting that K6- and K11-linked Ub chains positively regulate the mitophagy process (Durcan et al., 2014; Cunningham et al., 2015). Therefore, Parkin is considered a rather promiscuous E3 ligase that, once activated, ubiquitinates a broad range of proteins with a wide spectrum of Ub chains and amplifies the Ub signals via a positive-feedback manner to maximally ubiquitinated proteins on the damaged mitochondria.

Over 200 missense variants are identified in the *Parkin* gene, but only a small fraction has been clearly annotated to be pathogenic. Integrating clinical evidence with *in vitro* mitophagy activity, Yi et al. (2019) has conducted a systematic analysis of 51 Parkin variants, finding a correlation between the degree of mitophagy defect and the level of clinical manifestation. Among them, 13 Parkin variants are classified as pathogenic or likely pathogenic that all display severe mitophagy defects. Those variants designated as non-pathogenic show normal or near-normal mitophagy function (Yi et al., 2019). Likewise, Broadway et al. (2022) have investigated 10 rare Parkin mutants and found 7 with impaired mitophagy activity. These studies suggest that Parkin mediated mitophagy plays important roles in PD pathogenesis.

### 2.2 Parkin-mediated ubiquitination and mitochondrial-derived vesicles

In addition to mitophagy, an alternative mitochondrial quality control mechanism is via mitochondrial-derived vesicles (MDVs) (Cadete et al., 2016). With this, mitochondria transfer their defective mitochondrial contents to destination organelles, such as lysosomes, peroxisomes, and multivesicular bodies, for degradation via releasing vesicles with 70–150 nm in diameter (Futter et al., 1996; Soubannier et al., 2012a; Vasam et al., 2021; Rosina et al., 2022). Depending on the source of the mitochondrial stress, MDVs derive from the inner mitochondrial membrane (IMM) as a double-membrane structure or from the OMM as a single-membrane vesicle (Konig et al., 2021; Popov, 2022; Heyn et al., 2023). Whether Parkin plays a preferential role in the formation of MDVs remains to be elucidated. *In vitro*, the formation of MDVs carrying inner membrane marker PDH (pyruvate dehydrogenase) is dependent on Parkin activity (McLelland et al., 2014; Ge et al., 2020). Targeting of Parkin-dependent MDVs to the later endo-lysosomal compartments is mediated by the ternary SNARE protein complex composed of STX17 (syntaxin 17), SNAP29 (synaptosome associated protein 29), and VAMP7 (vesicle-associated membrane protein 7) (Soubannier et al., 2012a; Juhasz, 2016; McLelland et al., 2016). Unlike those PDH-positive MDVs, the presence of MDVs containing only OMM marker like Tom20 is not affected by loss-of-function Parkin mutants T240R or C431S, indicating the independence of Parkin E3 ligase activity (Soubannier et al., 2012b). However, Parkin was found to coordinate with Tollip (the endosomal adaptor Toll interacting protein) to facilitate the transport of these single-membrane MDVs to the endo-lysosomal compartment, with the help of the STX17-SNAP29-VAMP7 SNARE complex (Figure 1; Ryan et al., 2020). In contrast, Parkin seems to inhibit the formation of a subtype of MDVs that plays important roles in immune cells related mitochondrial antigen presentation (MitAP) through triggering ubiquitination and proteasomal degradation of SNX9 (sorting nexin 9) (Matheoud et al., 2016). Thus, Parkin-mediated ubiquitination potentially link between mitochondrial dysfunction and neuroinflammation in PD (Sliter et al., 2018). Consistently, Parkin-null mice show excessive inflammation due to STING-dependent (stimulator-of-interferon-genes dependent) pro-inflammation activity and increased vulnerability to inflammation-induced degeneration (Frank-Cannon et al., 2008; Sliter et al., 2018).

### 2.3 Parkin-mediated ubiquitination and other mitochondrial functions

#### 2.3.1 Parkin-mediated ubiquitination and mitochondrial dynamics

Mitochondria undergo constant fission-and-fusion in reflection of their functional statuses (Lewis and Lewis, 1914; Chen and Chan, 2017; Kraus et al., 2021). Increased mitochondrial fission results in small and round mitochondria to probably facilitate the mitochondrial trafficking within cells, while enhanced mitochondrial fusion leads to elongated mitochondria for efficient ATP production (Martini and Passos, 2023). Certain mitochondrial damages promote fission activity leading to the separation of depolarized mitochondria to facilitate their later removal by mitophagy or other mitochondrial quality control mechanisms (Han et al., 2020; da Silva Rosa et al., 2021; Cai et al., 2022).

**FIGURE 2:**
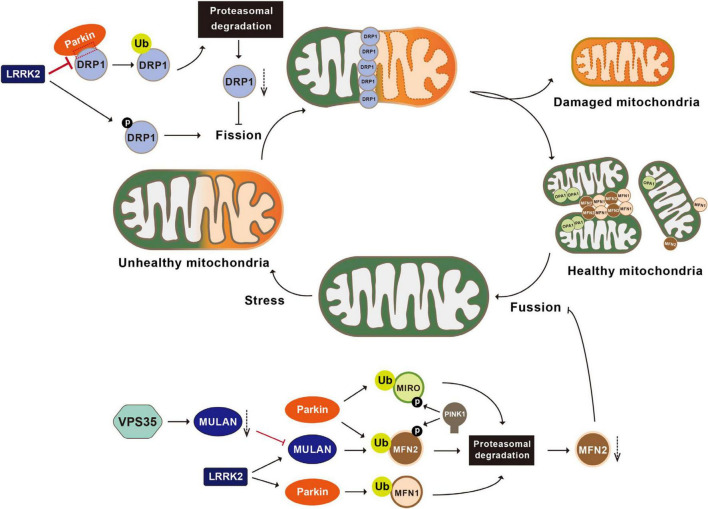
Post-translational modifications in the regulation of mitochondrial dynamics. Mitochondria undergo constant fusion (joining two mitochondria together with the help of MFN1, MFN2, and OPA1) and fission (separating one mitochondrion into two mitochondria with the help of DRP1). Parkin ubiquitinates DRP1, leading to the proteasomal degradation of DRP1 and inhibiting mitochondrial fragmentation. LRRK2 phosphorylates DRP1, impairs the interactions between Parkin and DRP1, and activates MULAN and Parkin’s E3 ligase activities, therefore regulating mitochondrial fragmentation. VPS35 regulates the degradation of the MULAN, thereby inhibiting MUL1 mediated ubiquitination and degradation of MFN2 and promoting mitochondrial fusion. Parkin ubiquitinates MFN1, MFN2 and MIRO, leading to their degradation via the proteasomal system and inhibiting mitochondrial fusion. PINK1 phosphorylates MIRO and activates Parkin mediated ubiquitination and degradation of MIRO. MFN1, mitofusin 1; MFN2, mitofusin 2; OPA1, optic atrophy 1; LRRK2, leucine-rich repeat kinase 2; VPS35, vacuolar protein sorting 35; MULAN, mitochondrial E3 ubiquitin protein ligase; MIRO, mitochondrial Rho GTPase.

Parkin is responsible for the ubiquitination and proteasomal degradation of several bona fide players in mitochondrial fission-and-fusion pathways, including the fission mediator DRP1 (dynamin-related protein 1) and fusion mediators MFN1 and MFN2 (Narendra et al., 2008; Yamada et al., 2018, 2019; Ham et al., 2020). Parkin interacts with DRP1 through its second RING domain and subsequently ubiquitinates DRP1, leading to its proteasomal degradation. Both the deficiency of Parkin and expression of the PD-associated Parkin mutant C431F inhibit ubiquitination and degradation of DRP1, resulting in mitochondrial fragmentation (Figure 2; Lutz et al., 2009; Wang H. et al., 2011). In *Drosophila*, overexpression of DRP1 rescues muscle degeneration and mitochondrial abnormalities in *PINK1*^–/–^ or *Parkin*^–/–^ mutants (Deng et al., 2008). *In vitro*, Parkin also ubiquitinates MFN-1 and MFN-2. Ubiquitination of MFN-1 and MFN-2 is reduced in either cell lines with Parkin deficiency or fibroblasts derived from PD patients harboring *Parkin* mutations (Gegg et al., 2010; Rakovic et al., 2011). In *Drosophila*, accumulated Marf (*Drosophila* homolog of MFN), along with reduced ubiquitinated Marf and increased non-ubiquitinated Marf, is observed in Parkin mutant flies (Poole et al., 2010; Ziviani et al., 2010). Thus, Parkin participates in regulation of mitochondrial dynamics.

#### 2.3.2 Parkin-mediated ubiquitination and mitochondrial biogenesis

**FIGURE 3:**
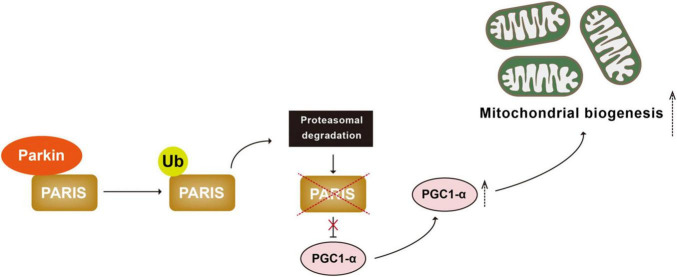
Parkin-mediated ubiquitination regulates mitochondrial biogenesis. PARIS binds to and represses PGC 1α, the master regulator of mitochondrial biogenesis. Parkin interacts with and ubiquitinated PARIS, leading to its proteasomal degradation and promoting mitochondrial biogenesis. PARIS, Parkin-interacting substrate; PGC 1α, peroxisome proliferator-activated receptor gamma coactivator 1-alpha.

Another mechanism cells employ to cope with mitochondrial damage is to generate new mitochondria, a process known as mitochondrial biogenesis (Ivankovic et al., 2016; Popov, 2020). Mitochondrial biogenesis is governed by the master regulator PGC1-α (peroxisome proliferator-activated receptor gamma coactivator 1-alpha). PGC1-α binds and actives nuclear transcription factors NRF-1 and 2 (nuclear respiratory factor 1 and 2) to increase transcription and therefore expression of proteins for mitochondrial biogenesis (Scarpulla, 2008; Li et al., 2011, 2021). PARIS (Parkin-interacting substrate) is a zinc finger protein that binds to and represses PGC-1α. Parkin interacts with PARIS and tags it with Ub chains for proteasomal degradation (Shin et al., 2011; Siddiqui et al., 2015; Stevens et al., 2015; Lee et al., 2017). Parkin deficiency leads to accumulation of PARIS, downregulation of PGC-1α, and selective loss of SNpc DA neurons. All of these are rescued by PGC-1α expression (Shin et al., 2011; Stevens et al., 2015; Siddiqui et al., 2016). These observations suggest that Parkin-mediated ubiquitination plays an important regulatory role in the PGC-1α-mediated mitochondrial biogenesis by ubiquitinating PARIS (Figure 3).

#### 2.3.3 Parkin-mediated ubiquitination and calcium homeostasis

**FIGURE 4:**
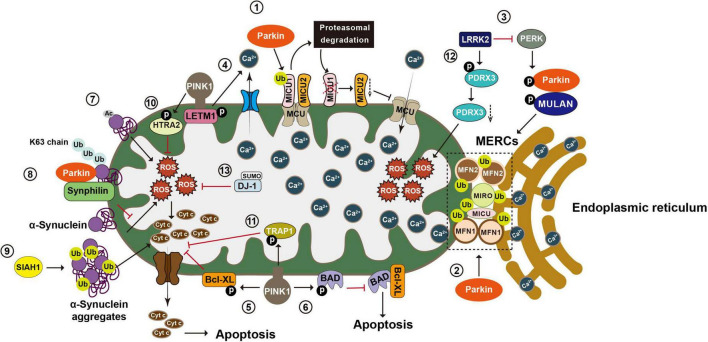
Post-translational modifications in the regulation of calcium homeostasis, oxidative stress, and apoptosis. Mitochondria play an essential role in maintaining Ca2+ homeostasis, regulating oxidative stress, and integrating apoptosis signals. (1) Mitochondria take up Ca2+ through the MCU complex, which is positively and negatively regulated by MICU1 and 2, respectively. Parkin regulates mitochondrial calcium homeostasis by directly ubiquitinating MICU1, leading to its proteasomal degradation, and indirectly affecting MICU2’s protein level. (2) Parkin also regulates calcium homeostasis by ubiquitinating tethering proteins involved in mitochondria endoplasmic reticulum contact sites (MERCs), such as MFN1, MFN2, MIRO1, and MICU. (3) LRRK2 blocks PERK mediated phosphorylation and activation of Parkin and MULAN, causing increased degradation of MERCS tethering proteins and reduced ER mitochondrial contacts. (4) PINK1 phosphorylates LETM1, leading to increased calcium release and facilitating calcium transport in mitochondria. (5) PINK1 phosphorylates Bcl XL and prevents its pro apoptotic cleavage. (6) PINK1 also phosphorylates BAD and prevents the formation of pro apoptotic Bcl XL BAD complex. (7) N α acetylation of α synuclein induces mitochondrial dysfunction. (8) Parkin increases K63-linked Ub chains on α synuclein, leading to the recruitment of Synphilin 1 to inhibit α synuclein toxicity. (9) SIAH-1 facilitates ubiquitination of α synuclein, increasing α synuclein’s insolubility and exacerbating its aggregation associated toxicity. (10) PINK1 mediated phosphorylation enhances the proteolytic activity of HTRA2 and protects cells against mitochondrial stress. (11) Phosphorylation of TRAP1 by PINK1 inhibits cytochrome c release and reduces cell death during oxidative stress. (12) LRRK2 phosphorylates PRDX3 and decreases its peroxidase activity. (13) SUMOylation of DJ 1 is crucial for of DJ 1’s anti-ROS activity. MCU, mitochondrial calcium uniporter; MICU1, mitochondrial calcium uptake 1; MICU2, mitochondrial calcium uptake 2; MFN1, mitofusin 1; MFN2, mitofusin 2; MIRO1, mitochondrial Rho GTPase 1; LRRK2, leucine-rich repeat kinase 2; PERK, protein kinase RNA-like ER kinase; LETM1, leucine zipper-EF-hand-containing transmembrane protein 1; ER, endoplasmic reticulum; Bcl XL, B-cell lymphoma-extra large; BAD, BCL2 associated agonist of cell death; Ub, ubiquitin; SIAH-1, seven *in absentia* homolog-1; HTRA2, high-temperature requirement serine protease A2; TRAP1, TNF receptor-associated protein 1; PRDX3, peroxiredoxin 3; ROS, reactive oxygen species.

Recent studies suggest that Parkin regulates calcium homeostasis by ubiquitinating a range of tethering proteins involved in mitochondria-endoplasmic reticulum contact sites (MERCs), such as MFN1, MFN2, MIRO1, and MICU (Figure 4). Parkin in calcium regulation was initially reported by Sandebring et al., 2009. With epidermal growth factor (EGF) stimulation, expression of both PD-associated Parkin mutants (R42P and G328E) or Parkin knockdown results in increased PLCγ1 (phospholipase C gamma1) and elevated basal cytosolic Ca^2+^ levels. These phenotypes are completely reversed with the treatment of PLC-inhibitor neomycin (Sandebring et al., 2009). A later study demonstrates that glutamate excitotoxicity in neuronal cells triggers Parkin accumulation on the ER and MERCs, suggesting a role of Parkin in the mitochondria-ER crosstalk (Van Laar et al., 2015). In Hela cells that do not express endogenous Parkin, overexpression of Parkin, but not the Parkin mutant lacking Ubl domain, enhances ER-mitochondrial tethering and increases agonist-induced Ca^2+^ transients (Cali et al., 2013). Conversely, Parkin knockdown impairs mitochondrial Ca^2+^ transfer and reduces mitochondria-ER contacts, suggesting that Parkin may enhance mitochondria-ER Ca^2+^ transfer by maintaining MERCs (Cali et al., 2013). However, confounding evidences are observed in fibroblasts derived from PD patients carrying *Parkin* mutations and in *Parkin*^–/–^ mice. Loss of Parkin results in close proximity between ER and mitochondria, leading to increased mitochondria-ER Ca^2+^ transfer, while overexpression of Parkin could restore the cytosolic Ca^2+^ transient to normal (Gautier et al., 2016).

Mitochondria take up Ca^2+^ through the mitochondrial calcium uniporter (MCU) complex that are composed of four core components, including the pore-forming subunit MCU, gatekeeping subunits MICU1 and 2 (mitochondrial calcium uptake 1 and 2), and the auxiliary subunit EMRE (essential mitochondrial regulator) (Sancak et al., 2013; Raffaello et al., 2013). MICU1 and 2, respectively, act as positive and negative regulators of the MCU complex (Plovanich et al., 2013). Under basal conditions, Parkin ubiquitinates MICU1, leading to its rapid degradation via the proteasomal system. Given that MICU2 stability is strictly dependent on MICU1, Parkin-mediated ubiquitination also indirectly regulates the amount of MICU2 (Matteucci et al., 2018). By maintaining appropriate levels of MICUs, Parkin plays a regulatory role in mitochondrial calcium homeostasis. Additionally, Parkin exerts its effects on ER-mitochondrial tethering via ubiquitinating of MFN2. ER located MFN2 forms a homotypic or heterotypic complex with either mitochondria-located MFN2 or mitochondria-located MFN1, thereby bridging mitochondria and ER (de Brito and Scorrano, 2008). Parkin ubiquitinates MFN2 on K416 in the HR1 domain, which in turn positively affects the physical and functional interactions between mitochondria and ER. Parkin-resistant MFN2 mutant K416R loses the ability to restore the decreased mitochondria-ER interactions in Parkin deficient cells and fibroblasts carrying PD-associated Parkin mutants (Basso et al., 2018). MIRO is another substrate of Parkin that regulates mitochondria-ER contacts. In yeast cells, yeast MIRO (Gem1p) deficiency leads to significant decrease of MERCs (Figure 4; Wang X. et al., 2011).

### 2.4 Ubiquitination of other PD-associated proteins and mitochondrial functions

#### 2.4.1 Ubiquitination of α-synuclein and mitochondrial functions

α-Synuclein, a principal component of LB, is a key protein involved both genetically and pathologically in PD. Mutations in the α*-synuclein* gene cause familiar forms of PD. The polymorphisms of α*-synuclein* confer a relatively increased risk of developing idiopathic PD. Since 1997, point mutations and duplication of α*-synuclein* gene have been inventoried with autosomal dominant PD, suggesting a gain-of-function mechanism (Polymeropoulos et al., 1997; Fuchs et al., 2007; Elia et al., 2013).

α-Synuclein is predominantly found in the presynaptic terminals of the central nervous system and is implicated in regulating synaptic plasticity and neurotransmitter release (Burre et al., 2010; Venda et al., 2010). Compelling evidence suggests that mitochondrial functions, such as cytochrome c release, calcium homeostasis, ATP production, and mitochondrial fission-and-fusion balance, are also directly regulated by α-synuclein. Remarkably, α-synuclein is found in all mitochondrial compartments and can selectively be localized to mitochondria under stress conditions (Ulmer et al., 2005; Li et al., 2007; Parihar et al., 2008; Zhang et al., 2008; Devi and Anandatheerthavarada, 2010; Subramaniam et al., 2014). The N-terminal 32 amino-acid sequence of α-synuclein is critical for its mitochondrial translocation. α-Synuclein undergoes multiple PTMs, including ubiquitination. α-Synuclein is a target of Parkin upon mitochondrial stress. Under basal conditions, no interaction between α-synuclein and Parkin is detected. With the treatment of CCCP or rotenone, Parkin forms a complex with α-synuclein and catalyzes formation of K63-linked Ub chains to recruit Synphilin 1, a negative regulator of α-synuclein toxicity, to the α-synuclein-Parkin complex. Therefore, Parkin-mediated ubiquitination may negatively regulate α-synuclein’s response to the mitochondrial stress (Norris et al., 2015). Consistently, α-synuclein is the substrate of RING-type E3 ligase SIAH-1 (seven *in absentia* homolog-1). SIAH-1 facilitates the mono- and poly-ubiquitination of α-synuclein to increase α-synuclein insolubility and exacerbate its aggregation-associated toxicity (Figure 4). Cells overexpressing WT α-synuclein or PD-associated mutant α-synuclein A53T show exacerbated cytochrome c release and apoptosis with increased of SIAH-1 activity (Lee et al., 2008).

Ubiquitinated α-synuclein is predominately found in LBs and has been demonstrated to contribute to the misfolding of α-synuclein, mitochondrial dysfunction, and neuronal death (Spillantini et al., 1997; Devi et al., 2008; Choubey et al., 2011). Despite these associations, efforts to establish α-synuclein as a biomarker for PD diagnosis or prognosis primarily focus on three species of α-synuclein-total α-synuclein, phosphorylated α-synuclein, and the oligomeric form of α-synuclein-in tissues and fluids like blood components, CSF, saliva, and extracellular vesicle (EVs) (Hong et al., 2010; Tokuda et al., 2010; Mollenhauer et al., 2011, 2013; Foulds et al., 2012; Wang Y. et al., 2012; van Steenoven et al., 2018; Vivacqua et al., 2019; Stuendl et al., 2021). Monoubiquitinated and polyubiquitinated α-synuclein are able to be detected in plasma using a polyclonal anti-ubiquitin antibody (FL-76) (Foulds et al., 2011). It will of interest in studying whether this is useful for PD diagnosis.

#### 2.4.2 FBXO7-regulated ubiquitination and mitochondrial functions

Mutations in *FBXO7* cause autosomal recessive EOPD (Shojaee et al., 2008). Being a F-box domain-containing protein, FBXO7 (F-box protein 7) acts as an adapter for Skp1-Cullin-F-box (SCF) type E3-ubiquitin ligase (Skowyra et al., 1997). Emerging evidence suggests that FBXO7 participates in the maintenance of mitochondrial homeostasis through facilitating ubiquitination modification of key proteins in the mitochondrial quality control pathways. Overexpression of FBXO7 enhances Parkin recruitment onto damaged mitochondria, suggesting a role of FBXO7 in mitophagy process (Burchell et al., 2013). It seems that FBXO7 negatively regulates mitophagy activity by enhancing the ubiquitination of PINK1, thereby, suppressing Parkin E3 ligase activity (Figure 1; Liu et al., 2020). Furthermore, ubiquitination of MFN1 was also found to be significantly reduced in both fibroblasts derived from patients harboring homozygous *FBXO7* R378G mutation and in SH-SY5Y cells with FBXO7 deficiency, which is restored by wild-type FBXO7. However, a recent study reports that FBXO7 is dispensable in the Parkin-mediated mitophagy process. Accumulation of pUb, recruitment of Parkin onto mitochondria, and mitophagy flux are all barely affected in *FBXO7*^–/–^ cells. Moreover, increased pUb foci were detected in *FBXO7*^–/–^ cells than that in control cells after treating with mitochondrial targeted HSP90 (heat shock protein 90) inhibitor Gamitrinib-TPP (Kraus et al., 2023). It is possible that FBXO7 regulates mitochondrial functions differentially depending on conditions. Nonetheless, the regulation is likely one via ubiquitination modification.

#### 2.4.3 VPS35-regulated ubiquitination and mitochondrial functions

VPS35 (vacuolar protein sorting 35) is a critical component of the retromer complex that is important for the retrograde transport of transmembrane protein-cargo from endosomes to the trans-Golgi network (TGN) (Hierro et al., 2007). Mutations in *VPS35* have recently been identified as the cause of familiar PD. A single missense mutation in *VPS35*, c.1858G > A (p.D620N), has been unambiguously identified to segregate with late-onset PD (LOPD) in an autosomal dominant manner (Zimprich et al., 2011). Studies suggest that VPS35 can indirectly participate in mitochondrial dynamics via the alteration of ubiquitination events. Using a transgenic mouse model, Tang et al. (2015) revealed that VPS35 regulates the trafficking and degradation of the MULAN (mitochondrial E3 ubiquitin protein ligase 1), thereby inhibiting MUL1-mediated ubiquitination and degradation of MFN2 and promoting mitochondrial fusion (Figure 2). Consistently, selective deletion of the VPS35 in DA neuron results in mitochondrial fragmentation and PD-relevant pathologies in *VPS35*^±^ mice (Tang et al., 2015).

#### 2.4.4 LRRK2-regulated ubiquitination and mitochondrial functions

Another PD-associated protein that indirectly participate in mitochondrial dynamics through ubiquitination modification is LRRK2 (leucine-rich repeat kinase 2). LRRK2, also known as dardarin (from the Basque word “dardara” that means trembling) and PARK8 (from early identified association with PD), is a large multifunctional kinase. Variants of this gene are associated with increased risk of PD and Crohn’s disease (Funayama et al., 2002). LRRK2, through its N-terminal domain, interacts with mitochondrial membrane-binding E3 ubiquitin ligases MARCH5 (membrane associated Ring-CH-type finger 5), MULAN, and Parkin. The kinase activity of LRRK2 is required for activation of these E3 ligase (Figure 2). No evidence of direct phosphorylation of these E3 ligases by LRRK2 has been found in *in vitro* assays, but screening assay using siRNA library revealed that PERK (protein kinase RNA-like ER kinase) is the kinase that directly phosphorylates and activates these E3 ligases. Via binding to these E3 ligases, LRRK2 WT blocks the PERK-mediated phosphorylation and activation of these E3 ligases. PD-associated LRRK2 mutant G2019S has decreased binding activity to these E3 ligases, resulting in increased phosphorylation and activation of E3 ubiquitin ligases by PERK, which consequently causes increased degradation of MERCS tethering proteins and reduced ER-mitochondrial contacts (Figure 4; Toyofuku et al., 2020).

## 3 Regulation of mitochondrial functions by phosphorylation in Parkinson’s disease

Protein phosphorylation, a prevalent PTM mediated by kinases, involves the covalent attachment of a phosphate group to an amino acid residue like serine (S), threonine (T), or tyrosine (Y). This dynamic modification offers a swift mechanism to alter protein function, thus playing crucial roles in regulation of various cellular pathways. Emerging evidence indicates that aberrant phosphorylation of proteins impacts mitochondrial functions, such as mitochondrial dynamics, mitophagy, MDV formation, mitochondrial respiratory activity, and calcium homeostasis. Notably, PD-associated kinase proteins, such as PINK1 and LRRK2, have been found to cause mitochondrial dysfunction through their dysregulated phosphorylation activity, ultimately leading to PD pathogenesis. Therefore, we aim to provide an overview elucidating how PINK1 and LRRK2 modulate a range of mitochondrial functions via their kinase activity, outlining their implications in the pathogenesis of PD through protein phosphorylation (Table 3).

**TABLE 3:** Phosphorylation of PD-related proteins and mitochondrial dysfunction.

PD-related protein	Enzyme	Substrate	Regulated mitochondrial function	Modification site (human)	References
PINK1	PINK1	PINK1	Activates PINK1	S228, S230, T257, S402	Kondapalli C. et al., 2012; Okatsu et al., 2012b; Aerts et al., 2015
	PINK1	Ub	Activates Parkin	S65	Kazlauskaite et al., 2014
	PINK1	Parkin	Promotes mitophagy, increases MDVs production	S65	Kazlauskaite et al., 2014
	PINK1	NDUFA10	Enhances CI activity	S250	Morais et al., 2014
	PINK1	Bcl-XL	Inhibits pro-apoptotic signal	S62	Arena et al., 2013
	PINK1	BAD	Prevents apoptosis	S112, S136	Wan et al., 2018
	PINK1	HTRA2	Reduces mitochondrial stress	S141	Plun-Favreau et al., 2007
	PINK1	TRAP1	Inhibits cytochrome c release	-	Pridgeon et al., 2007
	PINK1	LETM1	Maintains calcium homeostasis	T192	Huang et al., 2017
	MARK2	PINK1	Activates PINK1	T313	Matenia et al., 2012
LRRK2	LRRK2	Parkin	Inhibits mitophagy	-	Bonello et al., 2019
	LRRK2	MIRO	Inhibits mitophagy	-	Hsieh et al., 2016
	LRRK2	Rab10	Inhibits mitophagy	T73	Wauters et al., 2020
	LRRK2	BCL2	Increases mitophagy	T56	Su et al., 2015
	LRRK2	DRP1	Increases mitochondrial fragmentation	T595	Su and Qi, 2013
	LRRK2	4E-BP	Increases oxidative stress	T37, T46	Imai et al., 2008
	LRRK2	PRDX3	Increases oxidative stress	T146	Angeles et al., 2011
	LRRK2	unknown	Exacerbates mtDNA damage	-	Howlett et al., 2017; Gonzalez-Hunt et al., 2020

PINK1, PTEN induced putative kinase 1; Ub, ubiquitin; NDUFA10, NADH: ubiquinone oxidoreductase subunit A10; Bcl-XL, B-cell lymphoma-extra large; BAD, BCL2 associated agonist of cell eath; HTRA2, high-temperature requirement serine protease A2; TRAP1, TNF receptor-associated protein 1; LETM1, leucine zipper-EF-hand-containing transmembrane protein 1; MARK2, microtubule affinity-regulating kinase; LRRK2, leucine-rich repeat kinase 2; MIRO, mitochondrial Rho GTPase; BCL2, B-cell lymphoma 2; DRP1, dynamin-related protein 1; 4E-BP, 4E-binding protein; PRDX3, peroxiredoxin 3; MDV, mitochondrial-derived vesicle; CI, mitochondrial respiratory complex I; mtDNA, mitochondrial DNA; T, threonine; S, serine; “-” means no answer.

### 3.1 PINK1-mediated phosphorylation and mitophagy

Mutations in *PINK1* are the second most common cause of EOPD, accounting for 1–9% PD patients. More than 300 PINK1 variants have been identified from PD patients (Ma et al., 2021; Vizziello et al., 2021). The *PINK1* gene encodes a 581 amino acid protein, which contains an N-terminal mitochondrial targeting sequence (MTS), a transmembrane domain (TMD), and a highly conserved S/T kinase domain (Cardona et al., 2011). Under normal conditions, PINK1 is targeted to mitochondria through its MTS via the TOM (translocase of the outer membrane) and TIM (translocase of the inner membrane) complexes (Lazarou et al., 2012). During the translocation process, PINK1 undergoes consecutive cleavages by MPP (mitochondrial processing peptidase) and PARL (presenilin-associated rhomboid-like protease) (Jin et al., 2010; Deas et al., 2011; Greene et al., 2012), and the cleaved 52-kDa PINK1 is retro-translocated into the cytosol and undergoes rapid turnover by the proteasome via the N-end rule pathway (Whitworth et al., 2008; Greene et al., 2012). Therefore, PINK1 is normally maintained at a very low steady-state level. With damaged mitochondria, PINK1’s mitochondrial import is inhibited by the reduced mitochondrial membrane potential, which leads to the accumulation of full-length PINK1 on the OMM (Jin and Youle, 2013).

The landmark studies suggesting that both Parkin and PINK1 function through a common pathway to regulate mitochondrial function were from a series of *Drosophila* research (Greene et al., 2003; Clark et al., 2006; Park et al., 2006). Not only do *PINK1*^–/–^ and *Parkin*^–/–^ mutant flies exhibit similar degenerative phenotypes in neuron and muscle cells due to mitochondrial abnormalities, but overexpression of Parkin also rescues the *PINK1*^–/–^ phenotype, not vice versa, suggesting PINK1 acts upstream of Parkin in a linear pathway (Yang et al., 2006). Later on, more studies gradually unveil the models of how PINK1 and Parkin work together to regulate various mitochondrial functions (Durcan and Fon, 2015; Mouton-Liger et al., 2017).

PINK1-mediated phosphorylation plays essential roles at multiple steps of the mitophagy process, largely interacting with Parkin-mediated ubiquitination modification (Ordureau et al., 2014). First, autophosphorylation of PINK1 is important for its own activation, coinciding with its accumulation on the OMM (Kondapalli C. et al., 2012). PINK1 activity is determined by autophosphorylation at residues S228, S230, T257, and S402 (Kondapalli C. et al., 2012; Okatsu et al., 2012b; Aerts et al., 2015; Rasool et al., 2022). Although conflicting results regarding the regulatory role of autophosphorylation residues in PINK1are reported, autophosphorylation at S228 is important for the subsequent phosphorylation of PINK1 substrates in cells (Okatsu et al., 2012a; Aerts et al., 2015; Kumar et al., 2017; Rasool et al., 2018). Furthermore, the equivalent site of human PINK1 S228 is confirmed to be autophosphorylated in multiple PINK1 homologs, including S346 of *Drosophila* PINK1, S205 of *Tribolium* PINK1, and S202 of *Pediculus* PINK1 (Rasool et al., 2018). *Drosophila* carrying the PINK1 S346A mutant displays similar mitochondrial defects to those observed in *PINK1*^–/–^ mutant flies (Clark et al., 2006). Second, PINK1 phosphorylates Parkin at S65 of the ubiquitin domain to induce recruitment of Parkin to mitochondria and the release of Parkin E3 ligase activity. PINK1-mediated phosphorylation is essential for Parkin activation (Xiong et al., 2009; Kondapalli K. C. et al., 2012; Kane et al., 2014; Kazlauskaite et al., 2014; Koyano et al., 2014; Wauer et al., 2015a). In the absence of an activation signal, Parkin stays in the cytosol in an auto-inhibited structure due to the inhibitory intradomain contacts (Trempe and Gehring, 2023). PINK1 phosphorylates Ub on S65 (Kazlauskaite et al., 2014; Koyano et al., 2014; Wauer et al., 2015b). pUb serves as a receptor for Parkin to bind. Upon pUb binding, Parkin Ubl domain becomes more accessible by PINK1, leading to the subsequent phosphorylation of Ubl S65 (Durcan and Fon, 2015). Phosphorylation of Parkin by PINK1 further dissociates the inhibitory intradomain-contacting inside Parkin, resulting in the full activation of Parkin’s enzymatic activity (Trempe and Gehring, 2023). Third, PINK1-mediated phosphorylation amplifies the Parkin-mediated ubiquitination signal. After activation, Parkin ubiquitinates a large number of mitochondrial proteins and thereby produces increased Ub substrates for PINK1 to generate pUb signals, which successively initiate more Parkin recruitment and greater Parkin activation, creating a feed-forward loop to reach a maximal of Parkin-mediated ubiquitination (Figure 1; Ordureau et al., 2015; Koyano et al., 2019). On a relevant note, MARK2 (microtubule affinity-regulating kinase) is identified as an activating kinase of PINK1. MARK2 phosphorylates PINK1 at residue T313 that coincidentally is a residue frequently mutated to a non-phosphorylatable form T313M in PD cases (Tang et al., 2006). The expression of PINK1 T313M causes severe toxicity and abnormal mitochondrial accumulation in cells, also suggesting the mitochondrial consequence of the PINK1 activity (Matenia et al., 2012).

PINK1-mediated phosphorylation is a potential biomarker for PD diagnosis (Chin and Li, 2016). An antibody designed for pS65 on Ub (pS65-Ub) reveals that a rapid accumulation of pS65-Ub signal in mitochondria following mitochondrial damage. The presence of pS65-Ub positive granule also increases in cells derived from aging individuals and sporadic PD cases (Fiesel et al., 2015). Furthermore, a patent filed by Geldenhuys et al. (2014) details the use of PINK1 T257 autophosphorylation and PINK1-mediated phosphorylation of Parkin at S56 in serum and CSF as diagnostic measures. Additional efforts will be required to evaluate their clinical applicability.

Of the reported PD-associated *PINK1* mutations, about 30 of them are defined as “pathogenic” or “likely pathogenic,” causing similar clinical symptoms as in the cases caused by *Parkin* mutations (Richards et al., 2015; Ellard et al., 2020). Recently, Ma et al. (2021) analyzed 50 PINK1 variants and found most these pathogenic variants cause a significant decrease in mitophagy activity. However, these consist of only a small fraction of identified pathogenic PINK1. Further investigation is still needed to understand PINK1-phosphorylation regulated mitophagy in PD pathogenesis (Lin and Kang, 2008).

### 3.2 PINK1-mediated phosphorylation and mitochondrial-derived vesicles

PINK1, along with Parkin, are identified as key regulators of the MDV pathways that plays important roles in the regulation of mitochondrial turnover and MitAP production (Matheoud et al., 2016). PINK1 was initially found to be required for the formation of MDVs that deliver damaged mitochondrial portion to the lysosome for degradation (Sugiura et al., 2014; Pickrell and Youle, 2015). Ramirez et al. (2022) have recently reported that cannabidiol (CBD) activates PINK1 and Parkin in a dose-dependent manner, leading to elevated production of MDVs. CBD causes PINK1 accumulation on the mitochondrial out membrane to activate Parkin to promote the generation of MDVs (Figure 1). However, detailed mechanism underlying PINK1-regulated MDV formation remains unknown. It is possible that PINK1 participates in recognition of damaged sites of mitochondria and promotes segregation of damaged part of mitochondria. Consistent with this notion, PINK1 is shown to phosphorylate DRP1at S616 to activate fission, a potential mechanism to separate damaged and health portion of a mitochondrion (Han et al., 2020).

### 3.3 PINK1-mediated phosphorylation and other mitochondrial functions

#### 3.3.1 PINK1-mediated phosphorylation and mitochondrial dynamics

PINK1 is implicated in the phosphorylation of mitochondrial proteins that are important for the regulation of mitochondrial dynamics. First, PINK1 phosphorylates a group of key players in the mitochondrial dynamic pathways. The dynamin-related GTPase DRP1 is a crucial factor of the mitochondrial fission machinery, and its activity is regulated by phosphorylation (Yu et al., 2019; Han et al., 2020). Around 10 residues of DRP1 are able to be phosphorylated. Phosphorylation of S616 and S637 is extensively studied (Kim et al., 2016; Cha et al., 2021). After recruiting to the OMM, DRP1 is phosphorylated by PKA (protein kinase A) at S637, inhibiting DRP1 GTPase activity and suppressing its translocation to the mitochondria, and thereby impeding mitochondrial fission (Cereghetti et al., 2008). DRP1^*S*616^ was initially found to be phosphorylated by Cdk1/cyclin B, resulting in mitochondrial fragmentation (Pryde et al., 2016). We recently demonstrate that PINK1 directly phosphorylate DRP1^*S*616^ site to regulate mitochondrial fission that is independent of Parkin and autophagy activity (Han et al., 2020). MFN2 is also a substrate of PINK1. PINK1 phosphorylates MFN2 at residues T111 and S442, resulting in increased ubiquitination and proteasomal degradation of MFN2, leading to eventual mitochondrial fusion via Parkin mediated mechanism (Chen and Dorn, 2013; Tsai et al., 2014). Likewise, PINK1 phosphorylates MIRO1 at residue S156, in turn activates Parkin-mediated ubiquitination and degradation of MIRO1, therefore, inhibits axonal transport of mitochondria (Figure 2; Wang X. et al., 2011).

#### 3.3.2 PINK1-mediated phosphorylation and mitochondrial respiratory activity

Both PINK1 deficiency and PD-associated PINK1 mutants impair functions of mitochondrial respiratory complex I (CI) (Morais et al., 2009, 2014). NDUFA10 (NADH: ubiquinone oxidoreductase subunit A10) is an auxiliary subunit of CI. Although it is still unclear whether PINK1 directly phosphorylates NDUFA10, phosphoproteomic analysis reveals abolished phosphorylation of NDUFA10 at residue S250 in PINK1 knockout (KO) mice. Furthermore, both WT NDUFA10 and the phosphomimetic NDUFA10 mutant (S250D) enhance CI activity and rescue PINK1 deficiency-induced mitochondrial damage in mouse and cellular models. In contrast, the phosphorylation deficient mutant NDUFA10 S250A fails to rescue the PINK1 deficiency-related phenotypes (Morais et al., 2014). These results suggest a crucial role of phosphorylated NDUFA10 in the regulation of mitochondrial bioenergetics. Consistently, NDUFA10 improves PINK1 knockdown (KD)-induced mitochondrial hyperfusion in *Drosophila* by increasing CI activity (Pogson et al., 2014).

#### 3.3.3 PINK1-mediated phosphorylation and apoptosis

Mitochondria hold a central position in the apoptosis process. PINK1-mediated phosphorylation is found to prevent mitochondria-mediated cell death in multiple ways. PINK1 phosphorylates Bcl-XL (B-cell lymphoma-extra large) and prevents its pro-apoptotic cleavage. Bcl-XL has an anti-apoptotic activity by protecting the mitochondrial membrane potential (Δψ) and preventing cytochrome c release via its binding to and inhibition of VDACs. The N-terminal BH4 domain of the Bcl-XL is essential for this apoptosis inhibition activity (Shimizu et al., 2000). Upon mitochondrial depolarization, PINK1 interacts with Bcl-XL and phosphorylates it at residue S62, leading to the resistance of Bcl-XL to the cleavage of its N-terminal and the reduction of pro-apoptotic signal (Arena et al., 2013). BAD (BCL2 associated agonist of cell death) is a BH3-only protein. Phosphorylation of BAD at residue S112 inhibits its ability to form pro-apoptotic complex with Bcl-XL on the OMM (Hirai and Wang, 2001). Upon CCCP treatment, PINK1 phosphorylates BAD at residues S112 and S136, preventing the formation of pro-apoptotic Bcl-XL-BAD complex, leading to cell survival (Figure 4; Wan et al., 2018).

HTRA2 (high-temperature requirement serine protease A2) is implicated in the pathogenesis of PD and other neurodegenerative conditions. Mutations in *HTRA2* are a risk factor for sporadic PD cases. As a mitochondrial serine protease, HTRA2 functions in mitochondrial quality control and apoptosis. During apoptosis, HTRA2 is released into the cytosol and facilitates apoptosis by antagonizing IAPs (inhibitors of apoptosis). Interestingly, HTRA2 activation is dependent on the direct phosphorylation of its S141 by PINK1. PINK1-mediated S141 phosphorylation enhances the proteolytic activity of HTRA2 and protects cells against mitochondrial stress (Figure 4; Plun-Favreau et al., 2007). HTRA2 variants, A141S and P143A, identified in sporadic PD patients are in close proximity to the S142, suggesting they may contribute to PD by interfering with PINK1-mediated phosphorylation and HTRA2 activation (Strauss et al., 2005; Lin et al., 2011). Genetic studies in *Drosophila* further demonstrated the functional interaction between PINK1 and HTRA2. These studies collectively suggest that HTRA2, in parallel with Parkin, acts downstream of the PINK1 to maintain mitochondrial integrity (Whitworth et al., 2008; Tain et al., 2009). Consistently, reduced HTRA2 phosphorylation is observed in brains of PD patients carrying *PINK1* mutations (Plun-Favreau et al., 2007).

TRAP1 (TNF receptor-associated protein 1), also known as HSP75 (heat shock protein 75), is a mitochondrial chaperone protein. PINK1 binds to TRAP1 on mitochondria and phosphorylates TRAP1 in the mitochondrial intermembrane space (IMS). Phosphorylation of TRAP1 by PINK1 inhibits cytochrome c release and reduces cell death during oxidative stress. PD-associated PINK1 mutants G309D and L347P, both with reduced kinase activity, diminish the TRAP1 phosphorylation and result in increased apoptosis upon mitochondrial oxidative stress (Pridgeon et al., 2007). In *Drosophila*, TRAP1 deficiency results in mitochondrial dysfunction and vulnerability to various mitochondrial stress. Overexpression of human TRAP1 rescues PINK1-deficiency induced mitochondrial abnormalities (Costa et al., 2013; Zhang et al., 2013).

#### 3.3.4 PINK1-mediated phosphorylation and calcium homeostasis

Mitochondria are both major effectors and essential regulators of intracellular Ca^2+^ levels. The amount of Ca^2+^ retained inside the mitochondrial matrix is regulated by mitochondrial Ca^2+^ transient, which includes Ca^2+^ influx mediated by MCU and mitochondrial Ca^2+^ efflux mediated by Na^+^/Ca^2+^ and H^+^/Ca^2+^ antiporters (Szabadkai et al., 2006). PINK1 deficiency leads to dysfunction of the Na^+^/Ca^2+^ exchanger and causes mitochondrial calcium overload (Gandhi et al., 2009). Studies reveal that LETM1 (leucine zipper-EF-hand-containing transmembrane protein 1) is a mitochondrial H^+^/Ca^2+^ antiporter situated on the IMM. PINK1 interacts with LETM1 and directly phosphorylates it at residue T192, leading to increased calcium release in liposomes and facilitating calcium transport in mitochondria (Figure 4). Both PINK1 deficiency and PD-associated mutant PINK1 Q456X significantly reduce LETM1 phosphorylation, causing mitochondrial calcium-transport dysfunction and neuronal death (Huang et al., 2017).

### 3.4 LRRK2-mediated phosphorylation and mitochondrial functions

#### 3.4.1 LRRK2-mediated phosphorylation and mitophagy

Mutations in *LRRK2* are the most common cause of autosomal dominant LOPD (Zimprich et al., 2004). *LRRK2* encodes a 286-kDa protein containing multiple domains, including a leucine-rich repeat (LRR), a Ras of complex protein (ROC) GTPase domain, a mitogen-activated kinase domain, and WD40 domains. Therefore, LRRK2 is a bienzymatic protein with both GTPase and kinase activities. Six pathogenic mutations have been identified in *LRRK2*, including R1441C/G, N1437H, Y1699C, G2019S, and I2020T (Ross et al., 2011). The most common LRRK2 mutation, G2019S, is located right in the kinase domain. This mutation increases LRRK2 kinase activity toward itself and other substrates (Ross et al., 2011). Thus, the increased kinase activity of LRRK2 is considered important in the pathogenesis of PD.

While majority of LRRK2 is located at cytoplasm, a portion of LRRK2 is associated with the OMM (West et al., 2005; Biskup et al., 2006). iPSC-derived neural cells bearing LRRK2 G2019S and fibroblasts derived from PD patients carrying LRRK2 G2019S show mitochondrial impairment, suggesting that LRRK2 pathogenesis might involve mitochondrial dysfunction (Mortiboys et al., 2010; Sanders et al., 2014). *In vitro*, expression of LRRK2 increases mitochondrial clustering and reduced mitochondrial clearance upon CCCP treatment. In contrast, LRRK2 G2019S further exacerbated the damaging effects (Hsieh et al., 2016). Likewise, reduced mitochondrial autophagy in DA neurons and astrocytes in LRRK2 G2019S mouse brain that is rescued by treatment with LRRK2 kinase inhibitor GSK3357679A (Singh et al., 2021). Several possible mechanisms for the negative regulation of mitophagy by LRRK2 kinase activity are proposed: (1) LRRK2 impairs the interactions between Parkin and DRP1 and their mitochondrial targets in a kinase-dependent manner (Bonello et al., 2019); (2) LRRK2 interacts with MIRO, and the LRRK2 G2019S mutant prevents proteasomal degradation of MIRO, leading to delayed mitophagy (Hsieh et al., 2016); (3) LRRK2 phosphorylates Rab10 on the residue T73, and PD-associated LRRK2 mutants (G2019S and R1441C) impair Rab10 mitochondrial localization and disrupts its interaction with OPTN, resulting in impaired mitochondrial autophagy via a kinase activity related manner (Figures 1, 2; Wauters et al., 2020). In contrast, a study suggests a positive regulation of mitophagy by LRRK2’s kinase activity. LRRK2 G2019S phosphorylates BCL2 at residue T56, leading to the loss of Δψ and triggering excessive mitophagy via the recruitment of P62 to the mitochondria, (Su et al., 2015).

Recognizing the crucial role of elevated LRRK2 kinase activity in PD pathogenesis, multiple research groups have undertaken studies to quantitatively assess LRRK2-related phosphorylation across various tissues and biofluids, exploring their potential of being used as a PD diagnosis (Delbroek et al., 2013; Wang et al., 2017; Padmanabhan et al., 2020; Vissers et al., 2023). Collectively, the expressional level of total LRRK2 (tLRRK2), phosphorylation of LRRK2 at S1292 or S935 (pS1292-LRRK2 or pS935-LRRK2), and phosphorylation of the LRRK2 substrate Rab10 at T73 (pT73-Rab10) have been the focus of extensive study. The observed changes of tLRRK2 and LRRK2-associated phosphorylation in PD cases vary among brain regions, tissues, and cell types (Rideout et al., 2020). Increased tLRRK2 in the frontal cortex in sporadic PD cases, conflicting changes of tLRRK2 l in cerebrospinal fluid (CSF) in sporadic PD cases, elevated pS1292-LRRK2 in urinary EVs in but deceased pS935-LRRK2 in PBMCs among LRRK2-G2019S carriers, and increased pT73-Rab10 in neutrophils in idiopathic PD and LRRK2-G2019S carriers have been reported (Cho et al., 2013; Fraser et al., 2016; Fan et al., 2018; Mabrouk et al., 2020; Padmanabhan et al., 2020).

Remarkably, the highest expression of LRRK2 is not observed in neurons but in peripheral blood mononuclear cells (PBMCs) (Thevenet et al., 2011). Particularly, certain cell types in PMBCs show increased LRRK2 expression in PD patients compared to healthy controls, including B cells, T cells, CD16+ monocytes, neutrophiles, but not mixed PBMCs (Cook et al., 2017; Atashrazm et al., 2019). An LRRK2 inhibitor MLi2^25^ significantly reduced pS935-LRRK2 and pT73-Rab10 in both neutrophils and mixed PBMC (Atashrazm et al., 2019). Although pS935-LRRK2 does not directly reflect LRRK2 kinase activity like pS1292-LRRK2, it is sensitive to dephosphorylation caused by LRRK2 kinase inhibitor (Delbroek et al., 2013; Lobbestael et al., 2013). Therefore, pS935-LRRK2 and pT73-Rab10 are considered potential pharmacodynamics marker in clinical trials of LRRK2 kinase inhibitors.

#### 3.4.2 LRRK2-mediated phosphorylation and other mitochondrial functions

LRRK2 regulates mitochondrial dynamics via DRP1 in a kinase-dependent manner. In BV2 microglia cells and primary cultured microglia cells, treatment of lipopolysaccharide (LPS) activates microglia, resulting in enhanced mitochondrial fragmentation (Ho et al., 2018). Interestingly, LPS-induced mitochondrial fragmentation can be reversed by LRRK2 kinase inhibitor GSK2578215A. Results suggest an important role of LRRK2 kinase activity in regulating mitochondrial dynamics in microglia (Ho et al., 2018). Consistently, overexpression of WT LRRK2 or LRRK2 G2019S in cells results in apparent mitochondrial fragmentation, while overexpression of LRRK2 kinase-dead mutant D1994A does not cause such phenotype (Wang X. et al., 2012; Perez Carrion et al., 2018). LRRK2 directly interacts with and phosphorylates DRP1. PD-associated LRRK2 G2019S and R1441C mutants further enhance this interaction (Su and Qi, 2013; Stafa et al., 2014). LRRK2 G2019S phosphorylates DRP1 at residue T595 (Su and Qi, 2013). In fibroblasts derived from PD patients carrying LRRK2 G2019S, both the selective DRP1-inhibitor P110 or the expression of non-phosphorylatable mutant DRP1 T595A reverse the mitochondrial fragmentation and improve mitochondrial quality, indicating that LRRK2 G2019S-induced mitochondrial fragmentation is possible via a mechanism related to DRP1 T595 phosphorylation (Figure 2; Su and Qi, 2013).

Cells expressing PD associated LRRK2 mutants increase susceptibility to oxidative stress, suggesting that increased LRRK2 kinase activity might interfere antioxidant defense mechanism (Heo et al., 2010; Nguyen et al., 2011; Bahnassawy et al., 2013; Kim et al., 2019). LRRK2 phosphorylates 4E-BP (4E-binding protein) at residues T37/T46, both *in vitro* and *in vivo*. 4E-BP is a eukaryotic translation initiation factor regulating overall protein translation in cells that is crucial for cell survival under stress conditions (Haghighat et al., 1995; Tettweiler et al., 2005). *Drosophila* LRRK2 also phosphorylates 4E-BP, attenuating the resistance to oxidative stress via a 4E-BP phosphorylation-dependent manner (Imai et al., 2008). Therefore, LRRK2 kinase activity regulates cell response to oxidative stress through a 4E-BP mediated pathway. Studies also suggest that LRRK2 affects antioxidant defense mechanism through its phosphorylation of PRDX3 (peroxiredoxin 3). PRDX3 is a mitochondrial antioxidant of the thioredoxin-peroxidase family, efficiently scavenging peroxides and controlling the level of reactive oxygen species (ROS) in mitochondria (Fujii and Ikeda, 2002). *In vitro*, LRRK2 interacts with PRDX3 and potentially phosphorylates PRDX3 at residue T146. PD-associated mutant LRRK2 G2019S enhances its interaction with and causes decreased peroxidase activity of PRDX3 along with increased cell death (Figure 4; Angeles et al., 2011). Consistently, *Drosophila* expressing LRRK2 G2019S show reduced PRDX3 peroxidase activity and exacerbated oxidative stress (Angeles et al., 2014).

Furthermore, LRRK2 kinase activity is associated with increased mitochondrial DNA (mtDNA) damage. In various cellular models, including iPSC-derived neural cells, immune cells and fibroblasts, PD-associated mutant LRRK2 G2019S increases mtDNA damage that is abrogated by either gene editing to correct the G2019S mutation or by treatment with LRRK2 kinase inhibitors (Howlett et al., 2017; Gonzalez-Hunt et al., 2020).

Together, LRRK2-mediated phosphorylation regulates mitochondrial functions. Elevated LRRK2 kinase activity could contribute PD pathogenesis through impairing mitochondria.

## 4 Other post-translational- modifications: regulating mitochondrial functions in Parkinson’s disease

In addition to ubiquitination and phosphorylation, multiple other PTMs regulates mitochondrial functions and is implicated in the PD pathogenesis. In this context, we will provide a summary of recent research findings on how PD-related proteins affect mitochondrial functions through SUMOylation, acetylation, or s-nitrosylation, to elucidate their involvement in the development of PD (Table 4).

**TABLE 4:** Other PTMs of PD-related proteins and mitochondrial dysfunction.

Type of PTM	PD-related protein	Enzyme	Substrate	Regulated mitochondrial function	Modification site (human)	References
SUMOylation	DJ-1	/	DJ-1	Inhibits ROS production	K130	Shinbo et al., 2006; Krebiehl et al., 2010
	Parkin	/	Parkin	Increases Parkin’s ubiquitination	-	Um and Chung, 2006
Acetylation	PINK1	SIRT3	PINK1	Inhibits PINK1’s acetylation	-	Wei et al., 2017
	Parkin	SIRT3	Parkin	Inhibits Parkin’s acetylation	-	Wei et al., 2017
S-nitrosylation	Parkin	/	Parkin	Inhibits Parkin	-	Chung et al., 2004
	Parkin	/	Parkin	Activates Parkin	C323	Ozawa et al., 2013
	PINK1	/	PINK1	Inhibits PINK1	C568	Oh et al., 2017

PINK1, PTEN induced putative kinase 1; SIRT3, NAD-dependent protein deacetylase sirtuin-3; ROS, reactive oxygen species; C, cysteine; “-” means no answer.

### 4.1 SUMOylation-regulated mitochondrial function and Parkinson’s disease

SUMOylation refers to the PTM that covalently attaches small ubiquitin-like modifier (SUMO) to lysine residues on the substrate protein. SUMOylation occurs through multiple steps of enzymatic reactions, very similar to those in the ubiquitination process but with different specific enzymes, causing biochemical and functional changes of the target protein. SUMOylation modifies a broad range of proteins and regulates a diversity of biological processes, such as chromatin remodeling, transcription, and mitochondrial dynamics. SUMOylation regulates mitochondrial dynamics through a number of proteins that are either directly encoded by or in close functional-relationship with PD-associated genes (Guerra de Souza et al., 2016).

Mutations in *DJ-1* cause autosomal recessive forms of PD. Being a peroxiredoxin-like peroxidase, DJ-1 helps maintain mitochondrial function during oxidative stress as a sensor of damage and a regulator of CI activity (Taira et al., 2004; Andres-Mateos et al., 2007). DJ-1 deficiency results in increased production of ROS and decreased Δψ in cellular and mouse models (Krebiehl et al., 2010). SUMOylation plays important roles in regulating DJ-1 function. SUMOylation of DJ-1 K130 is crucial for the full activity of DJ-1 (Figure 4). The PD-associated DJ-1 L166P mutant becomes improperly SUMOylated and hence more insoluble, leading to its aggregation in mitochondria, ultimately suppresses its proteasomal degradation (Shinbo et al., 2006). DJ-1 is not only an effector of SUMOylation but also suppresses SUMOylating of other proteins at the global level through its interaction with key proteins of the SUMOylation machinery. For example, DJ-1 inhibits the SUMOylation of PSF (pyrimidine tract-binding protein-associated splicing factor), consequently reducing PSF-mediated apoptosis. The PD-associated pathogenic DJ-1 mutant L166P causes accumulation of high-molecular-weight SUMOylated PSF (Zhong et al., 2006). Together, these findings suggest that SUMOylation is involved in DJ-1’s regulation on mitochondria-associated oxidative stress and apoptosis.

Several biomarker studies have aimed to identify and quantify different DJ-1 species levels in PD patients compared to healthy controls. Presently, these studies primarily concentrate on the measurement of total DJ-1 in CSF, DJ-1 isoforms in whole blood, or oxidized DJ-1 in blood or urine (Waragai et al., 2006; Hong et al., 2010; Lin et al., 2012; Gui et al., 2015; Saito, 2017; Jang et al., 2018). Until now, the reliable detection of SUMOylated DJ-1 in various tissues or biofluids, and its potential use as an indicator for PD disease progression, stays unexplored. Similar to the challenge faced in using ubiquitinated proteins as biomarkers, the obstacle here may also be attributed to the lack of specific antibodies targeting SUMOylated DJ-1 (Magalhaes and Lashuel, 2022).

It’s noteworthy that not only is proper SUMOylation essential for the solubility and activity of DJ-1 protein, but also is the PKA induced phosphorylation at the T154 residue of DJ-1 (Ko et al., 2019). Currently, there is no evidence showing crosstalk between the T154 phosphorylation and the K130 SUMOylation of DJ-1. However, it is an intriguing question worth investigating.

Parkin has also been reported to selectively interact with SUMO-1 (small ubiquitin like modifier 1), both *in vitro* and *in vivo*, resulting in increased ubiquitination and nuclear translocation of Parkin. Therefore, SUMOylation might play a role in Parkin-mediated ubiquitination and its relevance to PD pathogenesis (Um and Chung, 2006). In agreement with this, DRP1 is a target of all SUMO isoforms with various functional-consequences. SUMOylation of DRP1 by SUMO-1 enhances its association with mitochondria, promotes mitochondrial fragmentation, and increases apoptosis (Wasiak et al., 2007). In contrast, SUMOylation of DRP1 by SUMO-2/3 decreases its mitochondrial localization and reduces apoptosis under stress conditions (Guo et al., 2013). Collectively, these studies underscore the significance of SUMO-regulated mitochondrial functions in the pathogenesis of PD.

### 4.2 Acetylation-regulated mitochondrial function and Parkinson’s disease

Protein acetylation refers to the transfer of an acetyl group (CH_3_CO) from acetyl-CoA to either the ε-amino group (NH_3_^+^) of lysine residues (ε-lysine acetylation) or to the N-terminal amino acid of a protein (*N*-α-acetylation). *N*-α-acetylation is an irreversible reaction catalyzed by N-terminal acetyltransferases (NATs), while l ε-lysine acetylation is a reversible modification tightly regulated by histone acetyltransferases (HATs) and histone deacetylases (HDACs) (Drazic et al., 2016). Recent evidence indicates that acetylation of PD-associated proteins might have important functional-consequences in mitochondria, although the detailed mechanism remains largely unknown.

*In vitro*, *N*-α-acetylation affects the secondary structure of α-synuclein, leading to the oligomeric form with a partial α-helical structure (Kang et al., 2012). *In vivo*, decreased Δψ and increased ROS level are detected in the mouse brain overexpressing predominantly N-terminally acetylated α-synuclein (Sarafian et al., 2013), suggesting that *N*-α-acetylation of α-synuclein cause mitochondrial dysfunction and have pathological implications in PD. Consistently, knockdown of deacetylase SIRT3 (NAD-dependent protein deacetylase sirtuin-3) in SH-SY5Y cells significantly increases rotenone-induced α-synuclein accumulation and reduces the activities of SOD (superoxide dismutase) and GSH (glutathione), leading to increased ROS generation and damaged mitochondria (Zhang et al., 2016). SIRT3 is also reported to be inversely related to the acetylation of Parkin and PINK1—acetylated PINK1 and Parkin are increased with knockdown of SIRT3 but decreased with overexpression of SIRT3 (Wei et al., 2017). PKAN (pantothenate kinase-associated neurodegeneration) is the enzyme catalyzing the first and rate-limiting step of CoA synthesis (Leonardi et al., 2005). A recently study reported that Fbl (Fumble, *Drosophila* homolog of PANK2), functioning downstream of PINK1, regulates acetylation of Ref(2)P (*Drosophila* homolog of P62) and promotes mitophagy activity (Huang et al., 2022).

### 4.3 S-nitrosylation-regulated mitochondrial function and Parkinson’s disease

S-nitrosylation involves the covalent attachment of a nitro oxide group (-NO) to the thiol side chain of a cysteine residue within a protein (Hess and Stamler, 2012). Like other PTMs, s-nitrosylation has emerged as an important regulator of various classes of proteins. S-nitrosylation plays a role in PD-related mitochondrial pathology through its modification of Parkin and PINK1. The initial two studies showing Parkin could be s-nitrosylated were both published in 2004, yielding contrary conclusions. Chung et al. (2004) reported that s-nitrosylation of Parkin inhibits its E3 ligase activity, resulting in decreased ubiquitination of Parkin substrates, including Parkin itself and Synphilin-1. While Yao et al. (2004) found that s-nitrosylation of Parkin stimulates its E3 ligase activity, leading to increased self-ubiquitination. Later on, Ozawa et al. (2013) reported that Parkin is predominantly s-nitrosylated at residue C323 resulting in activation of Parkin’s E3 ligase activity and induces mitochondrial degradation. Interestingly, s-nitrosylation of Parkin is regulated by DJ-1, another PD-associated protein that has been found in the same complex with Parkin and PINK1 (Tang et al., 2006; Xiong et al., 2009). Loss-of-function of DJ-1 results in decreased s-nitrosylation of Parkin, along with increased mitochondrial depolarization and cell death (Ozawa et al., 2020), adding additional evidence for the long-observed functional interaction between Parkin, PINK1, and DJ-1. Intriguingly, s-nitrosylation has also been observed with PINK1 at residue C568 that negatively regulates PINK1 kinase activity, therefore reducing PINK1-dependent phosphorylation and activation of Parkin (Oh et al., 2017). However, the detailed mechanisms of how s-nitrosylation regulates Parkin and PINK1 mediated mitophagy, as well as whether DJ-1 is also required for PINK1’s s-nitrosylation, remain unclear and require further study to elucidate.

## 5 Perspectives and conclusion

Mounting evidence indicates involvement of PTMs-regulated mitochondrial functions in the PD etiology. A major challenge in the field is to distinguish physiological functions from the pathological roles within these pathways implicated in PD. For example, understanding how PINK1/Parkin- and BNIP3-regulated mitophagy contributes to PD pathogenesis is crucial. It is well known that PINK1 and Parkin can function both collaboratively and independently regulating mitochondrial functions, but which function of PINK1 and Parkin is critical for PD? Moreover, since the deletion of PINK1, Parkin, or both does not result in significant DA neurodegeneration in mouse models, are there other factors important for DA neurodegeneration in patients carrying PINK1 and Parkin mutations?

Phosphorylated tau protein has recently been demonstrated as a valuable biomarker of Alzheimer’s disease at a systemic level (Karikari et al., 2020; Teunissen et al., 2022). A deeper understanding of the PD-related, specific alterations of PTMs-especially those with significant consequences on mitochondrial functions—may lead to candidates for the long sought-after biomarkers for PD. However, the development of PD biomarkers based on PTMs is currently in its early stages.

## Author contributions

SL: Writing – original draft, Writing – review & editing. DW: Writing – original draft, Writing – review & editing. ZZ: Conceptualization, Writing – review & editing.
